# Nighttime fluorescence phenotyping reduces environmental variability for photosynthetic traits and enables the identification of candidate loci in maize

**DOI:** 10.3389/fpls.2025.1595339

**Published:** 2025-05-26

**Authors:** Fangyi Li, Marcin Grzybowski, Rebecca L. Roston, James C. Schnable

**Affiliations:** ^1^ Department of Biochemistry, University of Nebraska-Lincoln, Lincoln, NE, United States; ^2^ Department of Agronomy and Horticulture, University of Nebraska-Lincoln, Lincoln, NE, United States; ^3^ Center for Plant Science Innovation, University of Nebraska-Lincoln, Lincoln, NE, United States; ^4^ Department of Plant Molecular Ecophysiology, Institute of Plant Experimental Biology and Biotechnology, Faculty of Biology, University of Warsaw, Warsaw, Poland

**Keywords:** photosynthesis, dark-adaptation, GWAS, genetic association mapping, maize

## Abstract

**Introduction:**

Photosynthesis is fundamental to agricultural productivity, but its relatively low light-to-biomass conversion efficiency represents an opportunity for enhancement. High-throughput phenotyping is crucial for unraveling the genetic basis of variation in photosynthetic activity. However, the heritability of chlorophyll fluorescence parameters measured during the day is often low as a result of high levels of variation introduced by environmental fluctuations.

**Methods:**

To address these limitations, we measured fluorescence phenotypes at night, leveraging natural dark adaptation to minimize environmental noise.

**Results:**

Night measurement significantly increased the heritability of fluorescence traits compared to daytime measurements, with the maximum quantum yield of photosystem II (Fv/Fm) showing an increase in heritability from 0.32 to 0.72. Genome-wide association studies (GWAS) conducted using three photosynthetic fluorescence traits measured at night across two growing seasons identified several significant single nucleotide polymorphisms (SNPs). Notably, two candidate genes near SNPs linked to multiple fluorescence traits, Zm00001eb271820 and Zm00001eb012130, have known roles in photosynthesis regulation. Four of the significant signal nucleotide polymorphisms identified in GWAS conducted using nighttime collected data also exhibited statistically significant associations with the same phenotypes during the day. In a majority of other cases, direction of effect was consistent but greater variance in day measured data relative to night measured data resulted in the differences not being statistically significant.

**Discussion:**

These results highlight the effectiveness of phenotyping photosynthetic traits at night in reducing environmental noise and enhancing the discovery of genomic intervals related to photosynthesis. While nighttime data collection may not be applicable for all photosynthetic traits, it offers a promising avenue for advancing our understanding of the genetic variation of photosynthesis in modern crop species.

## 1 Introduction

Photosynthesis captures light energy to create carbohydrates from carbon dioxide and water, thereby generating chemical energy for living organisms. While crops provide a primary source of food, feed, and fiber, the efficiency of photosynthesis remains relatively low, with a maximum of 6% of light energy being converted to stored chemical energy in the form of plant biomass (Zhu et al., 2008). Evolution optimized photosynthesis in the wild progenitors of modern crop plants for natural environments, but enhancing photosynthetic productivity in agricultural settings may be possible (Croce et al., 2024). Given current and projected food demands, boosting agricultural productivity is becoming more urgent (Pawlak and Kołodziejczak, 2020). Improvements to photosynthetic productivity could potentially increase agricultural production without a concomitant increase in requirements of land or agricultural inputs (e.g. fertilizer, water) (Zhu et al., 2010).

Light energy absorbed in the pigment antenna of photosystem II (PSII) can be channeled into three primary pathways: photochemistry, heat dissipation, or chlorophyll fluorescence (Butler, 1978). Photochemistry, the productive output of photosynthesis, uses the light energy to make reductant and ATP, which ultimately fix carbon dioxide. Excess light that cannot be captured by photochemistry is released as heat through multiple processes including both regulated, non-photochemical quenching (NPQ), and non-regulated mechanisms (PhiNO). Alternatively, excess energy can be emitted as chlorophyll fluorescence. These systems protect the plant from the highly reactive redox chemistry involved in light harvesting (Butler, 1978; Buchanan et al., 2015). By measuring the energy flux in any of these pathways, we gain insights into the overall efficiency and balance of photosynthesis (Maxwell and Johnson, 2000; Baker, 2008).

Since fluorescence emission data are easily captured optically, they have become common measurements from ecophysiology to basic science to applied agriculture (Maxwell and Johnson, 2000). By using a ‘modulated’ measuring system (Quick and Horton, 1984) multiple fluorescence parameters can be analyzed as part of the same experiment. The most commonly measured fluorescence-based parameter is the maximum quantum yield of PSII (*F_v_/F_m_*), which provides an estimate of photosynthetic efficiency (Fleischer, 1935; Kura-Hotta et al., 1987; Murchie and Lawson, 2013; Croft et al., 2017). Fluorescence parameters are also widely used as indicators of plant stress and tolerance. For instance, regulated (NPQ) and non-regulated (PhiNO) energy dissipation, along with functional energy use represented by *F_v_/F_m_*, are particularly informative parameters in studies of environmental abiotic stresses (Cornic and Briantais, 1991; Valentini et al., 1995; Niinemets et al., 1999; Sharma et al., 2017; Oakley et al., 2018; Itam et al., 2024).

Natural variation within populations for different photosynthetic parameters have been reported in a number of plant species (Flood et al., 2011; Hao et al., 2012; Strigens et al., 2013; Fiedler et al., 2016; Ortiz et al., 2017). Genome-wide association studies (GWAS) have identified genetic loci associated with variation in a range of photosynthesis related phenotypes, including genes involved in processes beyond photosynthesis itself, such as anatomical responses and central metabolism, in addition to identifying genes encoding components of the photosynthetic apparatus (Croce et al., 2024). However, few loci are consistently identified consistently across different studies, presumably due to differences in populations, standing functional genetic variation, environmental conditions, and experimental design. The repeated identification of new loci responsible for photosynthetic variation implies that there are many unrealized limitations on photosynthetic efficiency or productivity in many crop species.

To expand our understanding of genetic limitations of photosynthesis, we need more studies with strong associations collected in a diverse range of field-relevant environments. However, the heritability—or proportion of total phenotypic variation explained by differences between genotypes—of photosynthetic parameters tends to be relatively low, especially in field studies (Hao et al., 2012; Dramadri et al., 2021; Liu et al., 2023) because many photosynthetic parameters are responsive to even small environmental changes. While collecting data in controlled environments (e.g., greenhouses or growth chambers) can provide higher heritability (Strigens et al., 2013; Ortiz et al., 2017), in many contexts it can be unclear how well data from these controlled environments translate to the field. Attempts to control for field variability typically include extra treatments such as dark adaption, but these treatments create a trade-off between collection efficiency (Dramadri et al., 2021; Liu et al., 2023) and data quality (Araus et al., 1998; Hao et al., 2012).

Here, we investigated the potential for phenotyping photosynthetic florescence parameters in the field using a low-cost handheld fluorescence meter with and without night-induced dark-adaption. We collected measurements of multiple fluorescence traits from maize diversity panels across three years, including two years of data collected at night, and one year of data collected during the day. We used GWAS analysis to identify genetic markers associated with variation in photosynthetic fluorescence traits and explore the function of annotated genes in the genomic intervals surrounding those markers. We conclude that, with limitations, night measurements minimize environmental fluctuations during measurement, functionally increasing trait heritability and improving our ability to identify specific genomic intervals which explain variation in photosynthetic parameters in field environments.

## 2 Methods and materials

### 2.1 Field experiment

Field experiments were conducted at the University of Nebraska-Lincoln’s Havelock Research Farm (Lincoln, NE, USA) over three consecutive years, 2020 (40.85°N, 96.62°W), 2021 (40.51°N, 96.36°W), and 2022 (40.86° N, 96.60° W). In each year, maize diversity panels were grown in a randomized complete block design with two blocks. The field experiments included 752, 785 and 382 maize genotypes in 2020, 2021 and 2022 respectively. There were 665 common genotypes between 2020 and 2021, and 250 between 2021 and 2022. In 2020 and 2021, each block consisted of 840 plots (1,680 plots total), and 2022, each block consisted of 362 plots (724 total), with the majority of genotypes included once in each block and the remaining plots consisting of a repeated check genotype. Each plot consisted of two rows of 7.5 feet long with the spacing of 30-inch between each other, the spacing between two sequential plants was 4.5 inch, and the alleyways between two sequential plots was 30 inches. The planting dates were May 6^th^, May 7^th^, and May 20^th^ for 2020, 2021 and 2022.

### 2.2 Field data collection

Measurement of photosynthetic fluorescence traits was performed using a set of MultiSpeQ V2.0 instruments (Kuhlgert et al., 2016) running the Photosynthesis RIDES 2.0 protocol. In 2020, the photosynthesis fluorescence data was collected during the daytime from 09:00 to 14:30 over 4 days (July 23^rd^, 24^th^, 25^th^, and 28^th^) and used 6 instruments simultaneously as described (Ali et al., 2024). In both 2021 and 2022, photosynthetic fluorescence data was collected at night, with measurement beginning at 23:00, approximately 2 hours after sunset. Measurements were completed in 3 nights (August 5^th^, 6^th^, and 8^th^) using 6 instruments in 2021, and 1 night (August 4^th^) using 8 instruments in 2022. In all three years, the dates chosen corresponded to the period in which most plants in the field experiment were post flowering but had not yet reached the R5 (dent) stage. For each plot, 3 representative plants were semi-randomly selected, with the exceptions that lodged plants were always excluded and edge plants were excluded whenever sufficient numbers of non-edge plants were present. Measurements were collected from a fully expanded leaf. In 2021 and 2022 samplers were specifically instructed to score the leaf immediately above the first (uppermost) ear or ear shoot if present and intact. For night measurement, researchers involved in data collection employed green LED headlamps (~500–570 nm) to allow visibility while minimizing the disruption to photosynthesis, as green light has minimal overlap with the primary absorption peaks of chlorophyll a and b (430–450 nm and 640–680 nm, Taiz et al., 2015).

MultiSpeQ measurement of photosynthetic parameters followed the Pulse-Amplitude-Modulation (PAM) method (Schreiber, 2004), and obtained multiple fluorescence (*F*) values after stimulation of photosynthesis at different light inputs, including actinic illumination, fluorescence excitation, and far red (Kuhlgert et al., 2016). From MultiSpeQ outputs of *F*, we selected the following traits, maximum quantum efficiency (*F_v_’/F_m_’*), non-regulated energy dissipation (PhiNO), and the fraction of light dedicated to nonphotochemical quenching (PhiNPQ) (Genty et al., 1989; Kuhlgert et al., 2016).


Light Adapted Maximum Quantum Efficiency (Fv'/Fm')=(Fm'−F0')/Fm'

Non-regulatory Energy Dissipation (ΦNO)=Fs/Fm'

Fraction of light dedicated to NPQ (ΦNPQ)=1−ΦPSII–ΦNO

In 2021 and 2022, the natural dark adaptation of night, combined with the near-instantaneous light induction from the fluorometer suggested that the collected values of *F_m_’*, *F_v_’*, and *F_0_’* were equivalent to dark-adapted parameters, *F_m_*, *F_v_*, and *F_0_* (Schreiber, 2004). Specifically, under dark-adapted conditions, *F_0_* represents the minimal chlorophyll fluorescence yield when all PSII reaction centers are fully open, with primary quinone acceptor (Q_a_) fully oxidized (Maxwell and Johnson, 2000; Baker, 2008), a condition frequently achieved by night (e.g., Niyogi et al., 2001; Demmig-Adams et al., 2012). Similarly, *F_v_’*/*F_m_’* should approximate the maximum quantum yield of photosystem II (*F_v_*/*F_m_*) and the photosystem II operating efficiency (ΦPSII), while ΦNO and ΦNPQ should approximate their dark-adapted values (Schreiber, 2004).

### 2.3 Data processing and BLUP calculation

Empirically determined cutoffs based on manual examination of trait distributions were employed to remove extreme or biologically implausible values (**Supplementary Table S1**). We noted that in 2022, the range of *F_v_*/*F_m_* values included many lower values than in 2021 and the range of ΦNPQ included many higher values than in 2021, likely as a result of the more severe environmental stress experienced during the 2022 field season. Raw datasets for each trait were trimmed by removing values which did not meet the cutoff criteria described in **Supplementary Table S1**. After trimming of extreme values, BLUPs for each trait were calculated using the 2D spline based spatial correction model implemented in the SpATS R package (Rodríguez-Álvarez et al., 2018) with the identity of the specific MultiSpeQ instrument used to collect a given datapoint fit as an additional random effect (**Supplementary Table S3**). Generalized heritability for each trait in each environment was also calculated using the SpATS R package.

### 2.4 Genome wide association

The genetic marker data used for the genome-wide association studies (GWAS) described here were generated subsetting published genetic marker data generated using resequencing data of 1,515 maize lines (Grzybowski et al., 2023) relative to the B73_RefGen_v5 reference genome (Hufford et al., 2021). This marker set included data on 772 of the 785 maize genotypes planted in 2021 and 306 of the 382 maize lines planted in 2022. Two marker sets were created, one for 2021 and one for 2022, by using VCFtools (Danecek et al., 2011) to filter the published dataset to include only maize lines present in both the genetic marker and phenotype files and to include only single-nucleotide polymorphisms (SNPs), excluding InDels and other structural variants, with a minor allele frequency of at least 0.05 and a heterozygous genotype call frequency of 5% or less among those subsets of maize lines. These criteria resulted in subsets of 9,427,065 SNPs for 772 maize lines in 2021, and 9,672,830 SNPs for 306 maize lines in 2022. The effective number of independent tests represented by these two markers sets was estimated to be 2,774,165 for the 2021 marker set and 3,214,685 for the 2022 marker set via GEC (Li et al., 2012). GWAS analyses were conducted using the FarmCPU algorithm (Liu et al., 2016) as implemented in the R package rMVP (v.1.0.6; Yin et al., 2021), with the first three principal components calculated from the genetic marker data included as covariates. A bootstrap resampling procedure was employed to estimate the stability of significant associations (Valdar et al., 2009) in FarmCPU analysis. For every trait, 100 analyses were run, each using a randomly chosen 90% of phenotype values. SNPs were considered significantly associated in each bootstrap if they exceeded a Bonferroni corrected α=0.05 threshold based on the effective SNP number in each dataset, which corresponded to a p-value cutoff of 1.80 * 10–^8^ in 2021 and 1.56 * 10–^8^ in 2022. The resampling model inclusion probability (RMIP) for a given SNP was calculated by dividing the total number of iterations in which a given SNP was significantly associated with a trait of interest by the total number of iterations conducted.

### 2.5 Candidate gene exploration and marker effect validation

Genetic markers which exceeded an RMIP threshold of 0.1 were considered trait associated markers for the purposes of candidate gene exploration. Candidate genes were defined as those within ten kilobases up or downstream of the position of a trait associated marker. The significance of the phenotypic difference between sets of genotypes carrying different alleles of a given trait associated marker was assessed using unpaired two-tailed *t*-tests considering the BLUPs calculated for all maize genotypes homozygous for either the reference or alternative allele.

## 3 Results

### 3.1 Variation in photosynthetic fluorescence traits collected during the day or night

**Figure 1:**
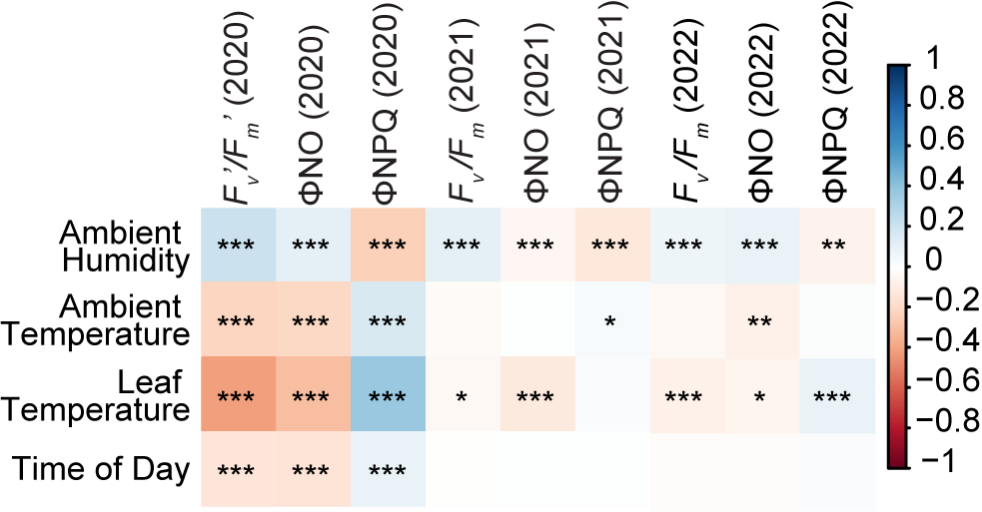
Correlations between environmental factors and photosynthetic fluorescence traits in datasets collected in different years. Pearson correlations were calculated between the values of environmental factors ambient humidity, ambient temperature, leaf temperature, and time of day, and the values of photosynthetic phenotype Fv’/Fm’ or Fv/Fm, ΦNO, and ΦNPQ in 2020, 2021 and 2022 datasets. All values for one single measurement were recorded simultaneously by MultiSpeQ. Intensity of the color to represent the strength and hue represents direction of the Pearson correlation coefficient (r). Red indicates a positive correlation. Blue indicates a negative correlation. *=P<0.05; **= <0.01; ***=P <0.001.

In 2020, a set of 5,053 daytime fluorescence measurements were collected of which with 4,940 measurements including at least one measurement of 746 genotypes remained after data quality control (see Materials and Methods; Ali et al., 2024). To compare these results with nighttime measurements, we collected similar data collection efforts in 2021 and 2022, resulting in 4,687 (of 772 genotypes) and 2,057 (of 306 genotypes) measurements after data control (**Supplementary Table S1**), where all the remaining genotypes have the genetic marker data for the GWAS analysis. Fluorescence traits measured during the day in 2020 were substantially correlated with variation in a number of environmental parameters including ambient humidity and temperature, leaf temperature, and time of day. However, for fluorescence traits measured at night in 2021 and 2022, correlations with these same environmental parameters were either substantially lower or absent (**Figure 1**). For example, the correlation between *F_v_’/F_m_’* and ambient temperature in 2020 was statistically significant, with a p-value below 0.05 and a correlation coefficient of -0.21, while *F_v_*/*F_m_* was insignificantly correlated with temperature in 2021 and 2022 (-0.026 and -0.036, respectively, **Supplementary Table S2**). In 2021, nighttime ambient temperatures ranged from 25.3 to 33.6°C, whereas daytime temperatures in 2020 ranged from 25.2 to 39.5°C. Other environmental factors also showed a similar narrower range during the night. This indicates that nighttime measurements relieved the most significant effects of measured environmental variation on photosynthetic traits.

To estimate the generalized heritability of fluorescence trait measurements collected in different years and environments, we first determined the best-performing model, defined as the one that controlled for the fewest factors – day, time of day, light, ambient temperature, leaf temperature, humidity, field spatial arrangement and variation in measurements across individual instruments (**Supplementary Table S3**) – while yielding the highest heritability. For the 2020 daytime measurements, the best-performing model accounted for the field’s spatial arrangement, instrument, day of measurement and leaf temperature. For the 2021 and 2022 nighttime measurements, the best-performing model controlled only for the spatial arrangement of the field and instrument. (**Supplementary Table S3**). Across all models, heritability of daytime measurements was consistently lower than nighttime measurements (**Supplementary Table S3**). In the best-performing models, nighttime heritability values ranged from 0.67 to 0.74 across years and traits, while daytime measurements ranged from 0.22 to 0.37 (**Table 1**).

**Table 1:** Generalized heritability of photosynthetic fluorescence traits for 2020, 2021, and 2022.

H_2_	F_v_’/F_m_’or F_v_/F_m_	ΦNO	ΦNPQ
2020, Day	0.12 (0.32)	0.35 (0.37)	0 (0.22)
2021, Night	0.72 (0.72)	0.69 (0.69)	0.74 (0.74)
2022, Night	0.7 (0.7)	0.74 (0.74)	0.67 (0.67)

Heritability (H_2_) values were estimated using the SpATS mixed model using the best performing models for night collected data and day collected data. The night model included genotype and the identity of the specific MultiSpeQ instrument as effects, while the day model additionally included day of collection and leaf temperature as additional effects.Values shown are heritability estimates from the night model, and the values in parentheses are the heritability estimates from the day model. *F_v_’*/*F_m_’* estimated maximum quantum yield of photosystem II, *F_v_*/*F_m_* maximum quantum yield of photosystem II, ΦNO quantum yield of non-regulated energy loss, ΦNPQ quantum yield of regulated non-photochemical energy loss.

### 3.2 Candidate gene identification for photosynthetic fluorescence traits

**Figure 2:**
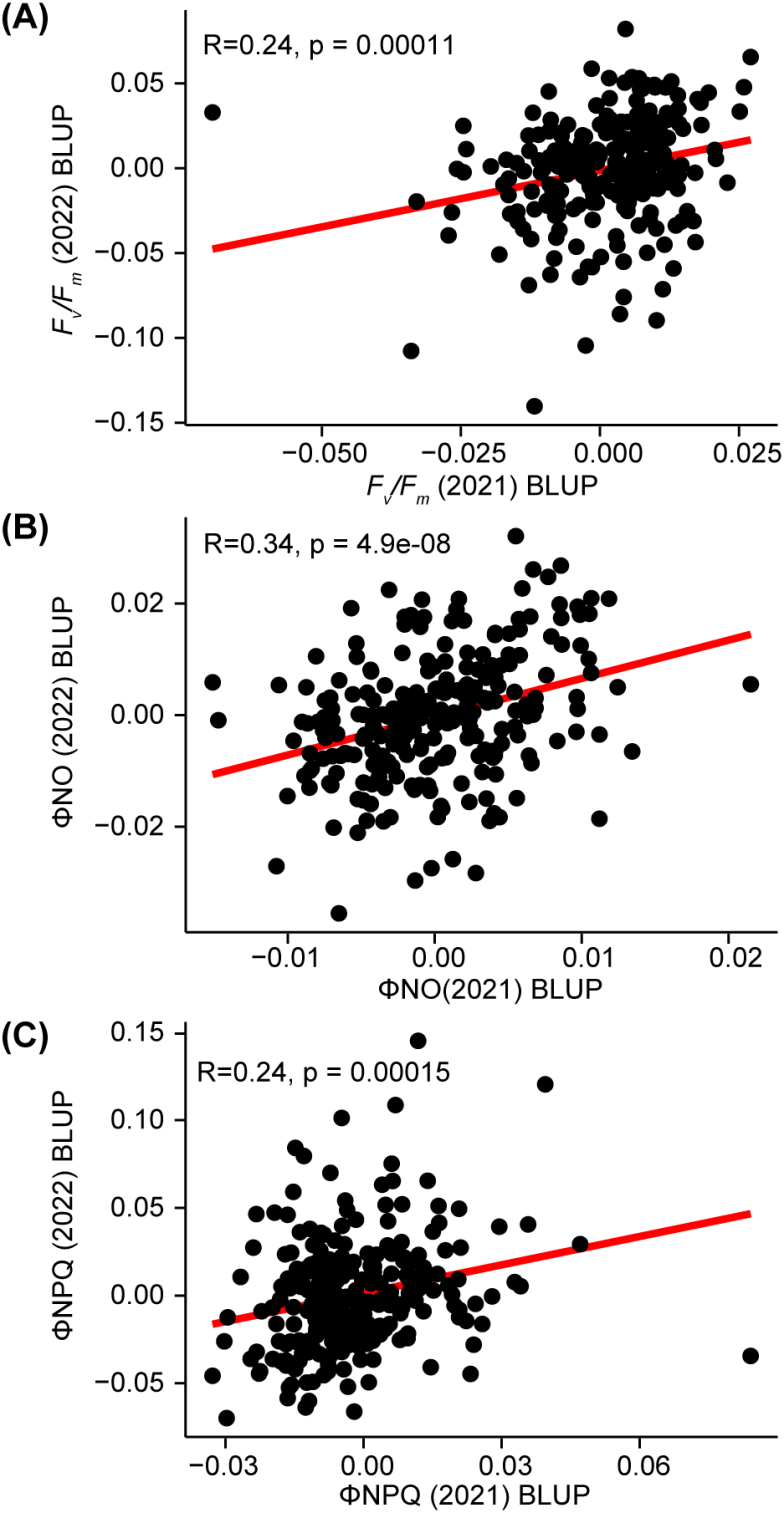
Correlation between photosynthetic fluorescence trait values measured for the same genotypes in 2021 and 2022. **(A)** Relationship between Fv/Fm BLUPs calculated for 2021 and 2022 for 250 maize genotypes phenotyped in both years. Sold red line indicates the regression line fit to this data. R = Pearson correlation coefficient p = statistical significance of the correlation across years. **(B)** Relationship between 2021 and 2022 ΦNO BLUPs. **(C)** Relationship between 2021 and 2022 ΦNPQ BLUPs.

Prior to conducting GWAS, we evaluated the consistency of photosynthetic fluorescence trait best linear unbiased predictor (BLUP) values for the same genotypes (n=248) scored during the night in two different years (2021 and 2022, **Figures 2A–C**). The most correlated trait between the two years was ΦNO, however, even for this trait the Pearson correlation coefficient was 0.34 (**Figure 2B**). This limited correlation observed across years indicated that, while genotypes exhibited some similarity in phenotype across years, substantial variation also existed between these two growing seasons. Indeed, stronger stress conditions in 2022 led to statewide decreases in corn yields relative to previous and subsequent years (USDA NASS, 2022 Census of Agriculture). Due to the limited similarities between seasons, we chose to perform separate GWAS analyses using data from each of the two years independently.

**Figure 3:**
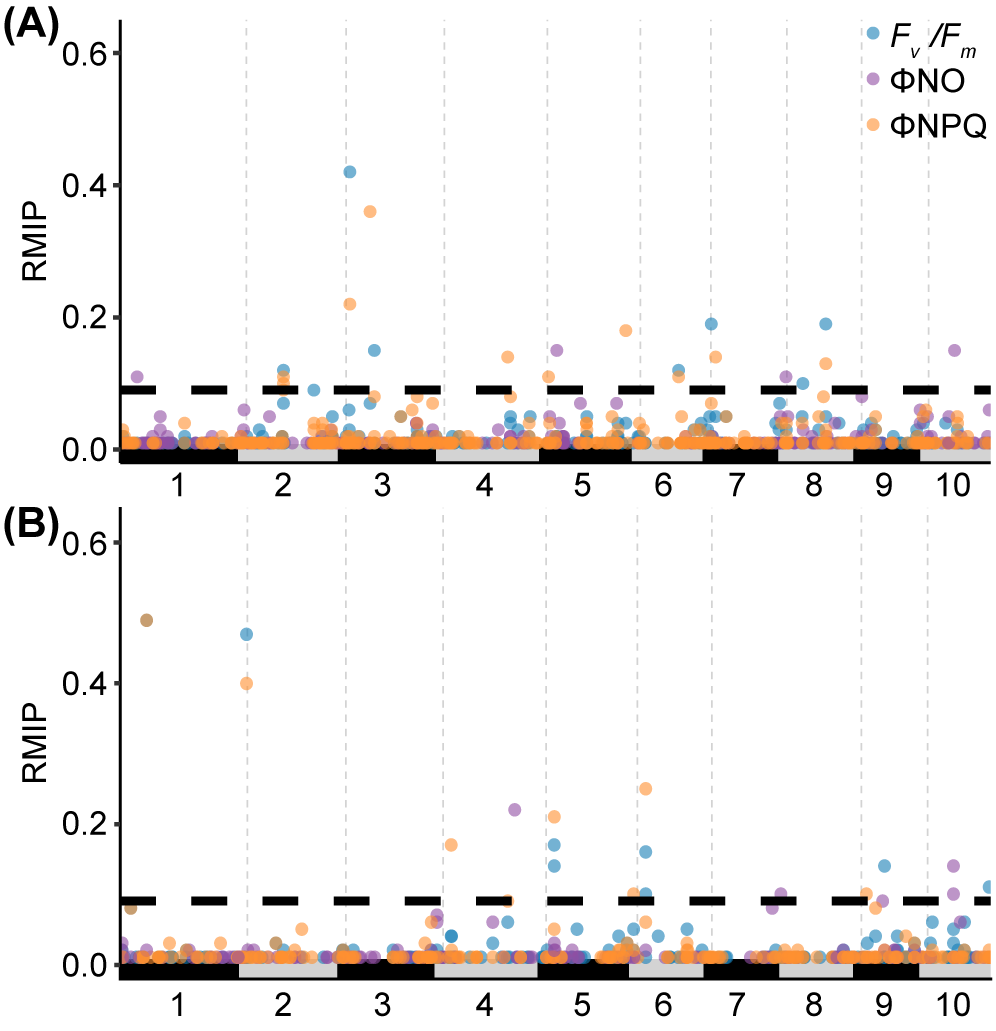
Results of resampling-based GWAS analysis of photosynthetic fluorescence traits in maize. **(A)** Analysis of genetic markers linked to variation in Fv/Fm, ΦNO and ΦNPQ using genetic marker and trait data for 772 maize genotypes grown in a replicated yield trial in Lincoln, NE in 2021. The position of circles on the x-axis indicates the position of a given marker on the maize genome pseudomolecules (B73: RefGen:V5). The position of circles on y-axis indicates the resampling model inclusion probability (RMIP) assigned to the same marker. The color of circles indicates the trait each marker is associated with. Only markers with a RMIP≥0.01 are shown. The maximum potential RMIP was 1.0. The y-axis is truncated at 0.65 to improve readability. Horizontal dashed line indicates the RMIP≥0.1 cutoff employed in this paper to prioritize markers with strong evidence of an association between the genomic interval and the trait(s) of interest. **(B)** Results of an equivalent GWAS conducted for the same three phenotypes using data from 306 maize genotypes collected in 2022.

Conducting GWAS with three photosynthetic traits scored in 2021 identified 22 SNPs which exceeded the confidence threshold (resampling model inclusion probability, RMIP, of greater than or equal to 0.1) (**Figure 3A**, **Supplementary Table S4**). Notably, two SNPs were associated with variation in all three photosynthetic fluorescence traits tested. Conducting GWAS with three photosynthetic traits scored in 2022 identified 15 SNPs associated with one or more traits that were past the confidence threshold (**Figure 3B**).

SNPs exceeding the confidence threshold were further investigated for their proximity to candidate genes potentially influencing photosynthesis. We chose to define genes within 10 kilobases of a trait-associated SNP as potential candidate genes based on the observation that average linkage disequilibrium in the Wisconsin Diversity panel decays below 0.25 between genetic markers separated by 10 kilobases (Grzybowski et al., 2023). A total of 17 genes near 12 trait-associated SNPs identified using trait data collected in 2021 met these criteria as did 11 genes close to 7 trait-associated SNPs identified from analysis of 2022 trait data (**Table 2**). Two genes with established roles in photosynthesis were located near significant SNPs identified in 2021. First, the SNP at chr01:39,614,496, associated with ΦNO (RMIP=0.11), was located 271 base pairs upstream of Zm00001eb012130, which encodes a chloroplast RNA polymerase sigma factor, ZmSIG2B, involved in light-dependent gene expression (Lahiri et al., 1999; Kanamaru et al., 2001; Fu et al., 2021). Second, the SNP at chr06:91,357,973, linked with both *F_v_*/*F_m_* (RMIP=0.12) and ΦNPQ (RMIP=0.11), was located 1,465 bp upstream of Zm00001eb271820, which encodes a protein involved in the stability of photosystem II (Chotewutmontri et al., 2020). The relevance of this gene to those phenotypes is demonstrated by the high chlorophyll fluorescence phenotype of the *hcf136* loss-of-function mutant in Arabidopsis (Meurer et al., 1998), and the reduction of photosystem II complexes in loss-of-function mutants of both maize and Arabidopsis (Plücken et al., 2002; Covshoff et al., 2008).

**Table 2:** Candidate genes close to significant SNPs.

Significant SNP	Trait (RMIP)	Year	Candidate Genes	Distance (bp)	NCBI Gene Description
chr1:39614496	ΦNO (0.11)	2021	Zm00001eb012130	271	sigma-like factor2B, RNA polymerase sigma factor
chr3:7881477	*F_v_/F_m_* (0.42); ΦNPQ (0.22)	2021	Zm00001eb121540	5822	AGC (cAMP-dependent cGMP-dependent and protein kinase C) kinase family protein.
chr5:1903250	ΦNPQ (0.11)	2021	Zm00001eb211140	2373	Biotin/lipoate A/B protein ligase family
chr5:188171960	ΦNPQ (0.18)	2021	Zm00001eb247160	2637	uncharacterized LOC100278954
chr6:91357973	*F_v_/F_m_* (0.12); ΦNPQ (0.11)	2021	Zm00001eb271810	3316	*no NCBI corresponding gene*
		2021	Zm00001eb271820	1465	photosystem II stability/assembly factor HCF136, chloroplastic
chr6:170168594	*F_v_/F_m_* (0.19)	2021	Zm00001eb292850	2813	O-methyltransferase ZRP4
		2021	Zm00001eb292870	3823	*no NCBI corresponding gene*
chr7:179748683	ΦNO (0.11)	2021	Zm00001eb329550	3823	uncharacterized protein family (UPF0114)
		2021	Zm00001eb329560	9103	uncharacterized LOC100384059
chr10:61529016	ΦNO (0.15)	2021	Zm00001eb413410	5366	Transcription factor GTE7
chr4:172155326	ΦNO (0.22)	2022	Zm00001eb190300	8959	*no NCBI corresponding gene*
chr7:165666120	ΦNO (0.1)	2022	Zm00001eb323840	1175	CYCD6
chr1:62661444	*F_v_/F_m_* (0.49); ΦNPQ (0.49)	2022	Zm00001eb017580	8745	glucose-1-phosphate adenylyltransferase large subunit 2, chloroplastic/amyloplastic-like
		2022	Zm00001eb017590	2458	probable low-specificity L-threonine aldolase 2
		2022	Zm00001eb017600	2615	uncharacterized LOC100281999
chr4:18671778	ΦNPQ (0.17)	2022	Zm00001eb169300	2776	Galactolipase DONGLE chloroplastic
chr5:18763852	*F_v_/F_m_* (0.17)	2022	Zm00001eb218900	9120	nucleotide-binding protein pseudogene
chr10:147695751	*F_v_/F_m_* (0.11)	2022	Zm00001eb432170	3740	cysteine protease 1
		2022	Zm00001eb432180	8025	*no NCBI corresponding gene*
chr9:54893176	*F_v_/F_m_* (0.14)	2022	Zm00001eb382440	4516	*no NCBI corresponding gene*
		2022	Zm00001eb382450	9969	*no NCBI corresponding gene*

The following information is provided for each candidate gene: Trait (RMIP), the resampling model inclusion probability (RMIP) value from GWAS associated with the corresponding fluorescence trait. Year, the year in which the SNP was identified in the GWAS analysis. Candidate gene, the name of the candidate gene(s) within 10 kb of the marker from the B73 genome (Zm-B73-REFERENCE-NAM-5.0: Zm00001eb.1). Distance(bp), the physical distance between the marker and the center of the gene coding region in base pairs. NCBI Gene Description, indicates the Gene description assigned to the gene by the National Center for Biotechnology Information. Traits are *F_v_*/*F_m_*, maximum quantum yield of photosystem II, ΦNO, quantum yield of non-regulated energy loss, and ΦNPQ, quantum yield of regulated non-photochemical energy loss.

### 3.3 SNP Effects on photosynthetic fluorescence traits across years

While significant trait-associated SNPs were identified via GWAS in both years, after multiple testing correction, no common signals were independently detected in both years, with the shortest distance separating 2021 and 2022 trait-associated SNPs exceeding 130kb. However, due to the stringent control for false positives and the millions of independent statistical tests conducted in GWAS a high false negative rate is expected (Sebastiani et al., 2009). To further investigate trait effects between years, we conducted single-locus tests, comparing BLUP values between individuals homozygous for the reference or alternative alleles of SNPs identified in one year across all years (**Table 3**). Despite not controlling for the effects of other loci elsewhere in the genome, as FarmCPU does, these simpler tests still identified statistically significant differences in the same trait and year of all but one SNP originally identified by GWAS (**Table 3**). Furthermore, for most trait-associated SNPs identified using nighttime measurements, the direction of the difference between reference and alternative alleles remained consistent across both daytime and nighttime measurements in different years (**Table 3**).

**Table 3:** Consistency of SNPs Effects Across Years.

SNP	REF	Trait	Year	2020(Day)	2021(Night)	2022(Night)
**chr2:88492028**	**T**	***F_v_/F_m_***	**2021**	- (*)	- (****)	- (*)
**chr8:37792190**	**G**	***F_v_/F_m_***	**2021**	- (**)	- (****)	- (*)
**chr7:179748683**	**G**	Φ**NO**	**2021**	- (**)	- (****)	- (*)
chr6:91357973	A	*F_v_/F_m_*	2021	- (ns)	- (****)	- (***)
chr8:93033455	C	*F_v_/F_m_*	2021	+ (ns)	+ (****)	+ (***)
chr1:39614496	C	ΦNO	2021	- (ns)	- (****)	- (*)
chr2:88492028	T	ΦNPQ	2021	+ (ns)	+ (****)	+ (*)
chr2:88496754	G	ΦNPQ	2021	+ (ns)	+ (****)	+ (*)
chr6:91357973	A	ΦNPQ	2021	- (ns)	+ (****)	+ (**)
chr8:93045879	G	ΦNPQ	2021	+ (ns)	+ (***)	+ (**)
chr5:188171960	G	ΦNPQ	2021	+ (*)	+ (****)	+ (ns)
chr3:7881477	T	*F_v_/F_m_*	2021	- (ns)	- (****)	- (ns)
chr5:21861350	G	ΦNO	2021	+ (ns)	+ (****)	+ (ns)
chr10:61529016	A	ΦNO	2021	- (ns)	- (ns)	- (*)
chr3:7881477	T	ΦNPQ	2021	- (ns)	+ (****)	- (ns)
chr4:151549745	C	ΦNPQ	2021	- (ns)	- (***)	- (ns)
chr5:1903250	T	ΦNPQ	2021	+ (ns)	+ (****)	+ (ns)
chr7:10158810	A	ΦNPQ	2021	+ (ns)	+ (****)	+ (ns)
chr5:18763852	T	*F_v_/F_m_*	2022	- (ns)	- (***)	- (***)
chr6:18990067	G	*F_v_/F_m_*	2022	- (ns)	+ (ns)	- (**)
chr6:18990114	T	*F_v_/F_m_*	2022	- (ns)	+ (ns)	- (**)
chr9:54893176	T	*F_v_/F_m_*	2022	- (ns)	- (ns)	- (*)
chr4:172155326	C	ΦNO	2022	- (ns)	- (ns)	- (***)
chr7:165666120	T	ΦNO	2022	+ (ns)	+ (ns)	+ (****)
chr10:61377705	A	ΦNO	2022	+ (ns)	+ (ns)	+ (****)
chr10:61394529	A	ΦNO	2022	+ (ns)	+ (ns)	+ (****)
chr4:18671778	A	ΦNPQ	2022	- (ns)	- (ns)	- (***)
chr5:210726575	C	ΦNPQ	2022	+ (ns)	+ (ns)	+ (**)
chr6:18990067	G	ΦNPQ	2022	+ (ns)	- (ns)	+ (**)
chr9:11153053	T	ΦNPQ	2022	+ (ns)	- (ns)	+ (***)

Summarized unpaired two-tailed t-tests comparing photosynthetic fluorescence phenotype best linear unbiased prediction (BLUP) values for individuals carrying either the reference or alternative alleles of significant SNPs present in both marker sets. SNP indicates chromosomal position. Bolded SNPs had the same direction of effect across all years, and all effects were significant. REF indicates the base of the reference allele. Traits are *F_v_*/*F_m_*, maximum quantum yield of photosystem II, ΦNO, quantum yield of non-regulated energy loss, and ΦNPQ, quantum yield of regulated non-photochemical energy loss. Year indicates the season each SNP was identified as significant by GWAS. In 2020, 2021, and 2022 columns, results of t-tests between fluorescence trait BLUPs comparing reference and alternative alleles within that year are reported. "-" or “+” indicate that individuals with reference alleles have higher or lower BLUP values, respectively. Significance is indicated by asterisks (*, p<0.05; **, p<0.01; ***, p<0.001). ns, non-significant.

Next, we considered markers which passed the separate inclusion criteria for both populations to identify year-to-year consistency of effect. For the 18 qualified SNPs identified in the 2021 GWAS analysis, 16 demonstrated the same direction of effect across all years. Among these, several also were significant effects in 2022, including four SNPs associated with *F_v_*/*F_m_*, two SNPs associated with ΦNO, and four SNPs associated with ΦNPQ. Notably, two SNPs associated with *F_v_*/*F_m_* and one SNP associated with ΦNO were significant across all years, including night and day measurements (**Table 3**). For the 12 qualified SNPs identified in the 2022 GWAS analysis, eight showed an identical direction of effect across all years, and one of these remained significant in 2021 (**Table 3**). These findings suggest that while some genetic variants have consistent effects on photosynthetic fluorescence across years, others are more influenced by environmental factors, such as daytime-induced factors in 2020 and the more extreme environmental conditions encountered in 2022.

## 4 Discussion

Phenotyping of photosynthetic traits across large populations is a powerful tool to identify genetic regions associated with photosynthetic activity, however, collecting comparable traditional daytime measurements from large populations in the field is often hindered by environmental fluctuations. This study explored the potential of nighttime fluorescence phenotyping as a more robust approach. By leveraging natural dark adaptation and minimizing environmental variability, nighttime measurements resulted in higher heritability values for fluorescence traits and stronger signals in the GWAS analysis (**Table 1**). Our study identified 37 SNPs associated with photosynthetic fluorescence parameters in a maize diversity panel over two field seasons (**Figure 3**, **Table 2**). Notably, two of these SNPs were located near genes with known roles in photosynthetic efficiency (**Table 2**), reinforcing the validity of nighttime phenotyping to identify genetic regulators of variation in photosynthetic traits. Additionally, several SNPs identified in 2021 were also associated with consistent differences in photosynthetic traits in the data collected in 2022, through consistent phenotypic trends in 2022 (**Table 3**), underscoring the reproducibility of our findings. These results further support the robustness of nighttime fluorescent phenotyping.

The challenge of measuring photosynthetic traits in comparable ways across large populations in the field is explained, at least in part, by the high sensitivity of photosynthesis to environmental factors. Nighttime measurements offered a more stable environment with a narrower range of ambient temperatures (25.3 to 33.6°C) and humidity (43.2% to 75.5%) compared to daytime (25.2 to 39.5°C and 36.7% to 75.4%). Additionally, the natural dark-adaptation inherent to nighttime conditions provides a standardized starting point for measurement across all samples and is a recommended method of dark adaptation (Kalaji et al., 2014). This is because during dark adaptation, photosystem II reaction centers return or remain in an ‘open’ state, ready to absorb light and initiate photosynthetic electron transport (Baker, 2008). Nighttime collection standardized all plants to this initial state, and the consistent modulated light protocol applied by the MultiSpeQ measuring instrument minimized the impact of varying light intensity during daytime collection. Light intensities vary meaningfully in field conditions as wind alters shadows cast by the plant and cloud cover, and varying light intensity has strong impacts on photoinhibition and photosynthetic efficiency (Alter et al., 2012; Han et al., 2023). While some instruments attempt to correct for this by using far-red light to approximate a measurement of *F_v_*/*F_m_* during daytime (e.g., LI-COR, MultiSpeQ used here), we observed relatively higher ΦNPQ and ΦNO values coupled with lower *F_v_*/*F_m_* in non-normalized fluorescence values in daytime versus nighttime measurements. This effect was consistent with that observed previously in pre-dawn measurements versus field daytime measurements (Demmig-Adams et al., 2012). These differences provide direct evidence that dark adaptation at night is a more consistent measurement of photosynthetic fluorescence, likely because of its effectiveness at resetting short-term energy dissipation strategies (i.e., NPQ and NO) and opening reaction centers.

Previous research reported heritability of photosynthetic fluorescence traits ranging from 0.08 to 0.38 in field experiments, with variations depending on the specific traits measured and phenotyping methods used. For *F_v_*/*F_m_* in Hao et al., 2012 using different chlorophyll meter; ΦNO and ΦNPQ in Dramadri et al., 2021 using MultiSpeQ; *F_v_’*/*F_m_’*, ΦNO and ΦNPQ in Liu et al., 2023 using MultiSpeQ. By minimizing environmental effects through nighttime measurement (**Figure 1**, **Supplementary Table S1**), we observed higher heritability values for fluorescence traits compared to daytime collection (**Table 1**). Higher heritability led to stronger signals in the GWAS analysis (**Figure 3**). Despite the smaller and only partially overlapping population phenotyped in 2022 and the challenging growth conditions in Nebraska that year (USDA NASS, 2022 Census of Agriculture), nearly half of the significant SNPs identified in the 2021 GWAS analysis replicated their effects on fluorescence phenotypes in 2022 (**Tables 2**, **3**).

### 4.1 Genetic factors contributing to maize photosynthetic parameters

The photosynthesis field has been aware of the value of night for dark adaptation for some time (Logan et al., 1999; Demmig-Adams et al., 2012). Intriguingly, it has been suggested that nighttime measurements are more likely to report longer-term adaptation responses (e.g., stress) more accurately than daytime measurements (Kalaji et al., 2014). In support of this idea, Nunes and colleagues compared pre-dawn and daytime measurements of *F_v_*/*F_m_* in cowpeas treated with drought or control conditions, finding that only pre-dawn measurements correlated with treatment (Nunes et al., 2022). We applied this relatively simple idea to a maize diversity panel to identify genes that might contribute to longer-term adaptation in our field environments. Among the two candidate genes with known links to photosynthesis, Zm00001eb271820, located on chromosome 6 and near the SNP marker, was significantly associated with *F_v_*/*F_m_* and ΦNPQ traits (**Table 2**), and is particularly noteworthy. This gene is named after its Arabidopsis homolog, whose mutant phenotype causes “high chlorophyll fluorescence”, HCF136 (Meurer et al., 1998). It was also identified and studied in maize through a transposon-tagging screen (Kolkman et al., 2005). Mutants lacking *HCF136* in maize and Arabidopsis have reduced levels of PSII complexes and altered thylakoid grana in their mesophyll chloroplasts (Meurer et al., 1998; Plücken et al., 2002; Covshoff et al., 2008). HCF136 directly affects the levels of PSII complexes through its effect on D1 assembly (Meurer et al., 1998; Covshoff et al., 2008) and synthesis (Chotewutmontri et al., 2020). This highlights the role of *HCF136* in maintaining photosystem II, providing a potential explanation for its association with the photosystem II traits *F_v_*/*F_m_* and ΦNPQ observed in our study (**Table 2**).

The second candidate gene, Zm00001eb012130, located on chromosome 1, was associated with the ΦNO trait (**Table 2**). The gene is named *Zm*SIG2B, and its function in maize is supported in part by its similarity to its Arabidopsis homolog (*At*SIG2), which encodes an RNA polymerase sigma factor (Lahiri et al., 1999). In plants, sigma factors regulate chloroplast gene expression and differentiation (Fu et al., 2021). Arabidopsis lacking *At*SIG2, *sig2-1*, had a pale-green phenotype and reduced PSII proteins (Kanamaru et al., 2001). Rather than directly affecting transcription of the genes encoding PSII components, *At*SIG2 is involved in transcription of plastid-encoded tRNAs essential for chloroplast development (Kanamaru et al., 2001; Fu et al., 2021). Additionally, subcellular fractionation and *in vitro* transcription assays have provided evidence that *Zm*SIG2B functions as a sigma transcription factor in maize chloroplasts (Beardslee et al., 2002). Expression analysis of closely related rice orthologs *Os*SIG2A and *Os*SIG2B, confirmed that these SIG2 homologs are involved in transcriptional regulation of chloroplast genes in rice (Kasai et al., 2004; Fu et al., 2021). Collectively, the evidence points to *Zm*SIG2B as a likely though indirect regulator of photosynthetic efficiency through its involvement in chloroplast transcription.

The SNPs identified in our GWAS analysis tend to exhibit consistent phenotypic effects across years, particularly for *F_v_*/*F_m_* and ΦNO traits (**Table 3**), which is likely to be related to the higher heritability in 2020 (**Table 1**). We examined if any SNPs overlapped with previous research employing a different measurement assay in the same maize population (Sahay et al., 2024). We did not identify any identical SNPs, however, one SNP associated with *F_v_*/*F_m_* in our study was located within a 50 kb interval containing 22 signals reported in that study, where the nearest signal is 83bp away from our SNP (**Supplementary Table S5**). In our study, this SNP also was one of the most consistent, with statistically significant similar effects across all three years of data employed in this study (**Table 3**).

### 4.2 Limitations of nighttime photosynthesis measurements

While nighttime fluorescence measurements offer several advantages for phenotyping, they also have certain inherent limitations. First, nighttime measurements cannot capture traits directly related to variable light environments, and other rapid environmental shifts experienced during the day (Kalaji et al., 2014). Second, there is a potential that the prolonged dark adaptation of night induces physiological changes that may influence measurements. Finally, nighttime is unlikely to be an ideal time to measure aspects of photosynthesis beyond fluorescence parameters, such as carbon assimilation. This is because carbon assimilation depends on stomatal opening, which increases at night (Caird et al., 2007). However, our data suggest that stomatal closure had little impact on fluorescence measurements. In C4 plants like maize, the separation of light and carbon fixation limits the influence of stomatal behavior, and dark adaptation further stabilizes the system. Consistently high *F_v_*/*F_m_*, low NPQ values, and stable environmental conditions across the field measurements(**Figure 1**, **Supplementary Table S2**)support the reliability of nighttime phenotyping in this context.

## 5 Conclusion

Our study demonstrates the advantages of nighttime fluorescence phenotyping for identifying genetic factors associated with a subset of photosynthetic traits. By minimizing environmental variability and providing a standardized starting point for measurements, nighttime phenotyping enhances the heritability of fluorescence parameters and reduces the influence of non-genetic factors. Despite the challenges posed by weather variations and the limitations of GWAS analysis, we identified multiple SNPs associated with photosynthetic traits. Notably, several of these SNPs are located near genes with known roles in photosynthesis, suggesting the potential for genetic improvement in this area. While nighttime phenotyping has certain limitations, our findings highlight its value as a tool for uncovering genetic regions related to photosynthesis. By incorporating nighttime phenotyping into breeding programs, researchers can accelerate the development of crop varieties with improved photosynthetic efficiency.

## Data Availability

The datasets presented in this study can be found in online repositories. The names of the repository/repositories and accession number(s) can be found in the article/**Supplementary Material**.
